# Diagnostic Performance of Core Needle Biopsy and Fine Needle Aspiration Separately or Together in the Diagnosis of Intrathoracic Lesions Under C-arm Guidance

**DOI:** 10.5334/jbsr.1615

**Published:** 2018-12-12

**Authors:** Youkyung Lee, Choong-Ki Park, Young-Ha Oh

**Affiliations:** 1 Department of Radiology, Hanyang University Guri Hospital, Hanyang University Collge of Medicine, KR; 2 Department of Pathology, Hanyang University Guri Hospital, Hanyang University College of Medicine, KR

**Keywords:** Lung biopsy, Fine needle aspiration, Core needle biopsy, C-arm cone-beam CT system

## Abstract

**Purpose:**

To evaluate and compare the diagnostic accuracy of fine needle aspiration (FNA) and core needle biopsy (CNB) of intrathoracic lesions using the same coaxial guide-needle under a C-arm Cone-Beam computed tomography system.

**Materials and Methods:**

Two hundred and eighty-eight patients (181 male, 107 female; 65.8 ± 13.3 years) with 293 lesions underwent 300 procedures, in which both FNA and CNB were performed. After inserting the coaxial guide-needle into the target lesion, we performed 18-gauge CNB, followed by 20-gauge FNA through the same coaxial guide-needle. The comparison of the procedures in which both showed adequate sample was performed with McNemar’s test (n = 229).

**Results:**

Of 300 procedures, 293 were technically successful. Adequate samples were obtained in 248/300 FNA and 288/300 CNB cases. The sensitivity and specificity for diagnosis of malignancy were respectively 84.7% (133/157), 100% (72/72) for FNA, when atypical cells included benign entity; 97.5% (153/157), 100% (72/72) for FNA, when atypical cells included malignancy; 97.6% (162/166), 100% (102/102) for CNB; and 100% (166/166), 100% (102/102) for combined FNA and CNB. Diagnosis of malignancy was significantly higher for CNB than for FNA (*p* < 0.001); however, it was not significantly higher when atypical cells included malignancy for FNA. Pneumothorax occurred in 50 (16.7%) and hemoptysis in 18 (6.0%) procedures.

**Conclusions:**

Combined use of CNB and FNA using the same coaxial guide-needle showed better diagnostic performance than using one alone. When comparing CNB and FNA, CNB showed significantly better performance, when atypical cells included a benign entity in FNA.

## Introduction

The image-guided percutaneous biopsy is a well-established diagnostic option for peripheral lung lesions [1]. Samples from fine needle aspiration (FNA) biopsy are used for cytological evaluation, whereas samples from core needle biopsy (CNB) are used for histologic evaluation. While there have been comparisons of FNA and CNB for diagnosing lung cancer, a comprehensive analysis of those studies is difficult, because the study designs are different [2,3]. Specifically, the studies use different image-guidance methods, usually CT or CT-fluoroscopy. Several studies compare two procedures performed on the same nodule in one visit [4,5,6,7,8,9], while others compare the groups in which one of the biopsy methods is used [10,11,12,13]. In some studies, the needle gauges used are not uniform, and coaxial guide-needles are not always used. Finally, an on-site pathologist is not always available [14,15].

In Hanyang University Guri Hospital, between December 2011 and March 2014, both FNA and CNB were routinely performed in the same procedure to enhance diagnostic accuracy. We used the same coaxial guide-needle for FNA and CNB, and the procedures were performed under C-arm cone-beam CT system (CBCT) guidance.

In this study, using the same coaxial guide-needle under CBCT guidance, we evaluated the diagnostic performance of the combined FNA and CNB in the diagnosis of intrathoracic lesions. In addition, we separately compared the diagnostic accuracy of FNA and CNB.

## Materials and Methods

Our institutional review board approved this retrospective study, and the requirement for informed consent was waived.

### Patients

This study included consecutive patients in a single institution who underwent CBCT-guided biopsies of intrathoracic lesions between December 2011 and March 2014. Of 334 total procedures, 34 biopsies in which both FNA and CNB were not performed together (22 FNA only, 12 CNB only) were excluded. This study included 300 biopsies of 293 intrathoracic lesions performed in 288 patients (181 male, 107 female; mean age, 65.8 ± 13.3 years). Five patients underwent two procedures for different pulmonary lesions. Ten patients underwent two procedures for the same pulmonary lesion, because of non-diagnostic biopsy results (n = 9) or molecular diagnosis (n = 1). One patient underwent three procedures for the same pulmonary lesion, again because of non-diagnostic biopsy results. In this study, repeated biopsies were considered separate initial procedures.

Physicians referred biopsy cases to the Radiology Department when a lung lesion was suspicious of lung cancer, or tuberculosis which may be hard to diagnose by sputum, bronchoscopic biopsy, or bronchoscopic lavage fluid.

We reviewed the medical records and images for the final diagnoses and identification of complications of the procedures.

### Biopsy and Aspiration Procedures

The procedures were performed by or under the supervision of a chest radiologist (C.P., 30 years of experience in image-guided lung biopsy). CNB and FNA procedures were performed using a CBCT system with one plane mode (Artis Zee Biplane, 30 cm × 40 cm flat panel detector, Siemens Healthcare, Erlangen, Germany). Pre-procedural CBCT images were transferred to dedicated workstations (Syngo Leonardo with DynaCT, Siemens Healthcare). Computed tomography (CT) images were reconstructed using multiplanar reformations in the axial, coronal, and sagittal planes. Using a virtual guidance program (Syngo iGuide, Siemens Healthcare), the chest radiologist determined the effective needle pathway to the target lesion. This image-guided procedure has previously been described [16,17].

After a 17-gauge coaxial guide-needle was inserted into the target lesion, one or two core biopsies were achieved with an 18-gauge standard semi-automatic core needle (Stericut, TSK Laboratory, Tochigi-Ken, Japan) through the coaxial guide-needle. After retrieval of the cutting core needle, an FNA was performed using a 20-gauge needle (Franseen Biopsy Needle, Angiotech Medical Device Technologies, now Argon Medical Devices, Gainesville, Florida, USA) through the same coaxial guide-needle. No on-site pathologist was available to verify the adequacy of the cytological or core sample specimen. Core specimens were immersed in 10% formalin for pathologic examination. Aspirated specimens were smeared on slides, which were then immersed in a 95% ethyl alcohol solution. The needle and syringe were rinsed with normal saline and sent for cytological evaluation. For diagnosis, the core specimen underwent hematoxylin-eosin stains and appropriate immunohistochemical stains.

To identify any pneumothorax, a fluoroscopic spot image in the supine position was obtained just after the biopsy. Chest radiographs of all patients were obtained 4 hours and 24 hours post procedure.

### Assessment of Diagnostic Performance

In the present study, technical success was determined by retrieval of an adequate sample from either FNA or CNB on pathologic examination.

FNA was reported according to the following classes in our institution: Class 0, insufficient or inadequate for diagnosis; Class I, negative for malignancy; Class II, benign; Class III, suspicious malignant (atypical cells); Class IV, highly suspicious for malignancy; and Class V, positive for malignant cells with a specific type. This classification was adapted from the diagnostic categories for pulmonary cytopathology [18].

**Figure 1:**
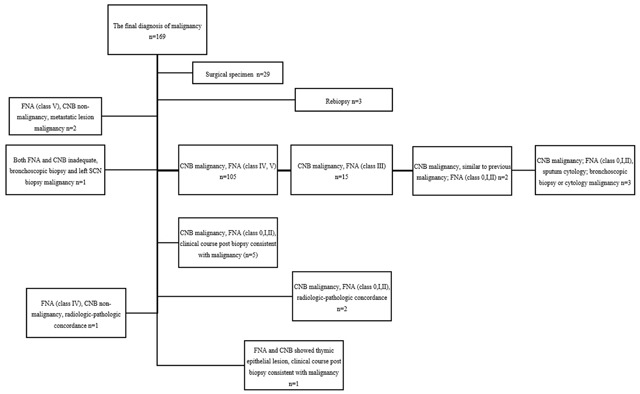
The final diagnosis of malignancy. Note. FNA-fine needle aspiration biopsy. CNB-core needle biopsy. SCN-supraclavicular lymph node.

A final diagnosis of malignancy was made when a surgical specimen showed malignancy (n = 29), or when the specimen showed malignancy in both CNB and FNA (class IV, V) (n = 103), and the results are shown in Figure 1.

A final diagnosis of a benign lesion (n = 104) was made (a) when a surgical specimen showed benign lesion (n = 2); (b) when the specimen showed benign-specific lesions on pathologic analysis, including benign tumors (n = 12), tuberculosis (n = 33), fungal infection (n = 11), cytomegalovirus associated infection (n = 2), actinomycosis (n = 3), and eosinophilic granulomatosis with polyangiitis consistent with clinical finding (n = 1); (c) when tuberculosis polymerase chain reaction result of the specimen showed *Mycobacterium Tuberculosis* (n = 2) or when sputum culture showed *Mycobacterium Tuberculosis* (n = 1). When nonspecific-benign findings such as inflammation, necrotic debris, eosinophilic pneumonia, granulomatous inflammation, organizing pneumonia, or eosinophilic pneumonia were present in FNA or CNB, lesions were considered benign only when they decreased (n = 9) or disappeared (n = 27), or when they were stable in size for at least two years (n = 1) [5].

Intrathoracic lesions that did not satisfy a final diagnosis of malignant or benign were considered ‘indeterminate,’ and were excluded from the calculation of diagnostic accuracy (n = 27). The samples which showed inadequate in both FNA and CNB were considered technically unsuccessful and were excluded from the calculation of diagnostic accuracy (n = 7; final diagnosis: 3 malignant, 2 benign, 2 indeterminate). The sensitivity, specificity, and overall diagnostic accuracy of malignancy in the FNA, CNB, and combined FNA and CNB samples were calculated. FNA results were presented in two ways: (a) when Classes IV and V were considered malignant (atypical cells considered benign), and (b) when Classes III, IV, and V were considered malignant (atypical cells considered malignant). If the results of FNA and CNB contradicted each other, then the result that matched final diagnosis was recorded as a result of combined FNA and CNB. If one sample was adequate and one was inadequate, then the result of an adequate sample was recorded as a result of combined FNA and CNB.

A specific cell type in malignancy was defined as a specific type of malignancy, including adenocarcinoma, squamous cell carcinoma, and metastasis from an organ other than the lung. Non-small cell carcinoma was defined as no specific cell type in malignancy.

### Comparison of FNA and CNB

Comparison of FNA and CNB was performed where both procedures showed an adequate sample (229 procedures). Comparison of diagnosis of a specific cell type of malignancy was performed in 157 procedures where malignancy was present. Comparison of diagnosis of benign-specific lesions was performed in 72 procedures that were benign. McNemar’s test was used for comparison of FNA and CNB. MedCalc Statistical Software version 16.4.3 (MedCalc Software bvba, Ostend, Belgium; https://www.medcalc.org; 2016) was used for the analyses. The significance level was set at *p* < 0.05.

## Results

Of 300 procedures, 293 (97.6%) were technically successful. Adequate samples were obtained in 248/300 (82.7%) FNA cases and 288/300 (96.3%) CNB cases. Both FNA and CNB samples were adequate in 229/300 (76.3%) procedures.

Of the technically successful procedures, the final diagnosis was malignant in 166/293 (56.8%) cases, benign in 102/293 (34.6%) cases, and indeterminate in 25/293 (8.6%) cases.

The types of malignant lesions are found in Table 1.

**Table 1:** Results of 166 malignant lesions.

Diagnosis	No. of cases	(%)

Adenocarcinoma	90	(54.2)
Squamous cell carcinoma	39	(23.5)
Small cell carcinoma	14	(8.4)
Metastatic adenocarcinoma	4	(2.4)
Pleomorphic carcinoma	3	(1.8)
Large cell carcinoma	2	(1.2)
Thymoma	2	(1.2)
Metastatic melanoma	2	(1.2)
Others	10	(6.0)

Others are the diagnoses with fewer than 2 cases.

**Figure 2:**
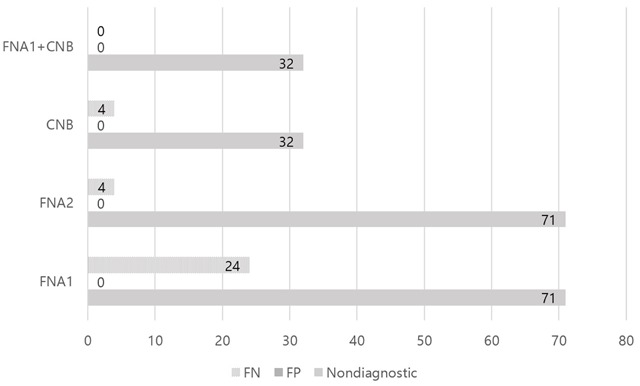
Comparison of FNA or CNB. Note. FNA = fine needle aspiration. In FNA1, atypical cells classified as benign. In FNA2, atypical cells classified as malignant. CNB = core needle biopsy. Nondiagnostic means inadequate sample or indeterminate of final diagnosis. FN = false negative. FP = false positive.

The overall sensitivity, specificity, and accuracy were respectively 84.7% (133/157), 100% (72/72), and 89.5% (205/229) for FNA, when atypical cells were classified as benign; 97.5% (153/157), 100% (72/72), and 98.3% (225/229) for FNA, when atypical cells were classified as malignant; 97.6% (162/166), 100% (102/102), and 98.5% (264/268) for CNB; and 100% (166/166), 100% (102/102), and 100% (268/268) for combined FNA and CNB (Table 2, Figure 2).

**Table 2:** Result of FNA or CNB.

Result	No. (%) of thoracic lesions			
	FNA (atypical cells classified as benign)	FNA (atypical cells classified as malignant)	CNB	FNA (atypical cells classified as benign) + CNB

True-positive	133 (44.3%)	153 (51.0%)	162 (54.0%)	166 (55.3%)
True-negative	72 (24.0%)	72 (24.0%)	102 (34.0%)	102 (34.0%)
False-positive	0 (0)	0 (0)	0 (0)	0 (0)
False-negative	24 (8.0%)	4 (1.3%)	4 (1.3%)	0 (0)
Nondiagnostic	71 (23.7%)	71 (23.7%)	32 (10.7%)	32 (10.7%)

*Note.* FNA = fine needle aspiration, CNB = core needle biopsy. Nondiagnostic means inadequate sample or indeterminate of final diagnosis. N = 300.

Among 52 inadequate FNA samples: eight were indeterminate, 32 were benign, and 12 were malignant; seven were also inadequate in CNB. Among the 12 malignant lesions which showed an inadequate sample in FNA, two malignant lesions were also inadequate in CNB.

Among four false negative samples by CNB, two were adenocarcinoma and the other two were squamous cell carcinoma in FNA. In one of the cases of adenocarcinoma, CNB showed infarct. In one of the cases of squamous cell carcinoma, CNB showed necrotizing squamous cells in necrotic background. In the other two false negative CNB, the samples showed normal lung parenchyma.

**Figure 3:**
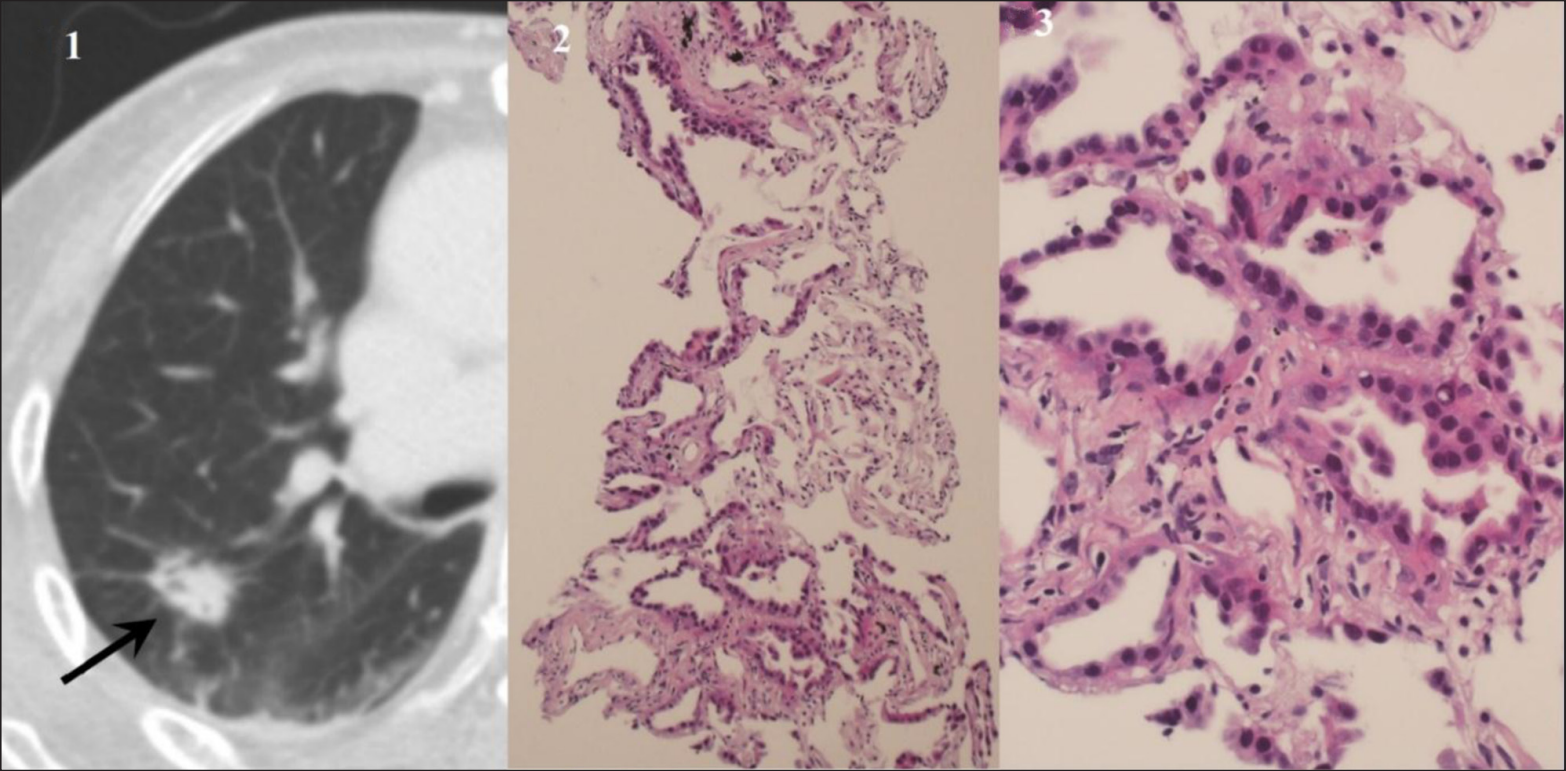
An adenocarcinoma with lepidic pattern diagnosed using samples from core needle biopsy. Diagnosis of fine needle aspiration was highly suspicious for adenocarcinoma. **(1)** CT image shows a nodule with inner bubble-like lucency in the right upper lobe (arrow). **(2)** Photomicrograph of a core biopsy (hematoxylin-eosin, original magnification × 100) shows lepidic growth along the alveolar interstitium with preserved alveolar architecture. **(3)** Magnified photomicrograph (hematoxylin-eosin, original magnification × 400) shows characteristic nonmucinous lepidic adenocarcinoma.

We assessed the diagnostic performance of the procedures in which both FNA and CNB showed an adequate sample (n = 229). One hundred fifty-seven cases were malignant. The overall sensitivity, specificity, and accuracy were respectively 84.7% (133/157), 100% (72/72), and 89.5% (205/229) for FNA, when atypical cells were classified as benign; 97.5% (153/157), 100% (72/72), and 98.3% (225/229) for FNA when atypical cells were classified as malignant; and 97.5% (153/157), 100% (72/72), and 98.3% (225/229) for CNB (Table 3). The diagnosis of malignancy was significantly higher for CNB than for FNA (*p* < 0.001). When we included atypical cells in malignancy for FNA, the diagnosis of malignancy was not significantly different between CNB and FNA (*p* > 0.05). Six cases were FNA class IV (Figure 3), and 127 were FNA class V. The specific cell types in malignancy were classified in 81.5% of the specimens obtained by FNA (128/157), whereas 96.8% were classified in CNB (152/157). The rate of specific cell type determination in malignancy was significantly higher for CNB than for FNA (96.8% vs. 81.5%, *p* < 0.001).

**Table 3:** Result of both FNA and CNB adequate sample.

Result	No. of thoracic lesions			
	FNA (atypical cells classified as benign)	FNA (atypical cells classified as malignant)	CNB	FNA (atypical cells classified as benign) + CNB

True-positive	133	153	153	153
True-negative	72	72	72	72
False-positive	0	0	0	0
False-negative	24	4	4	4

*Note.* FNA = fine needle aspiration, CNB = core needle biopsy, N = 229.

**Figure 4:**
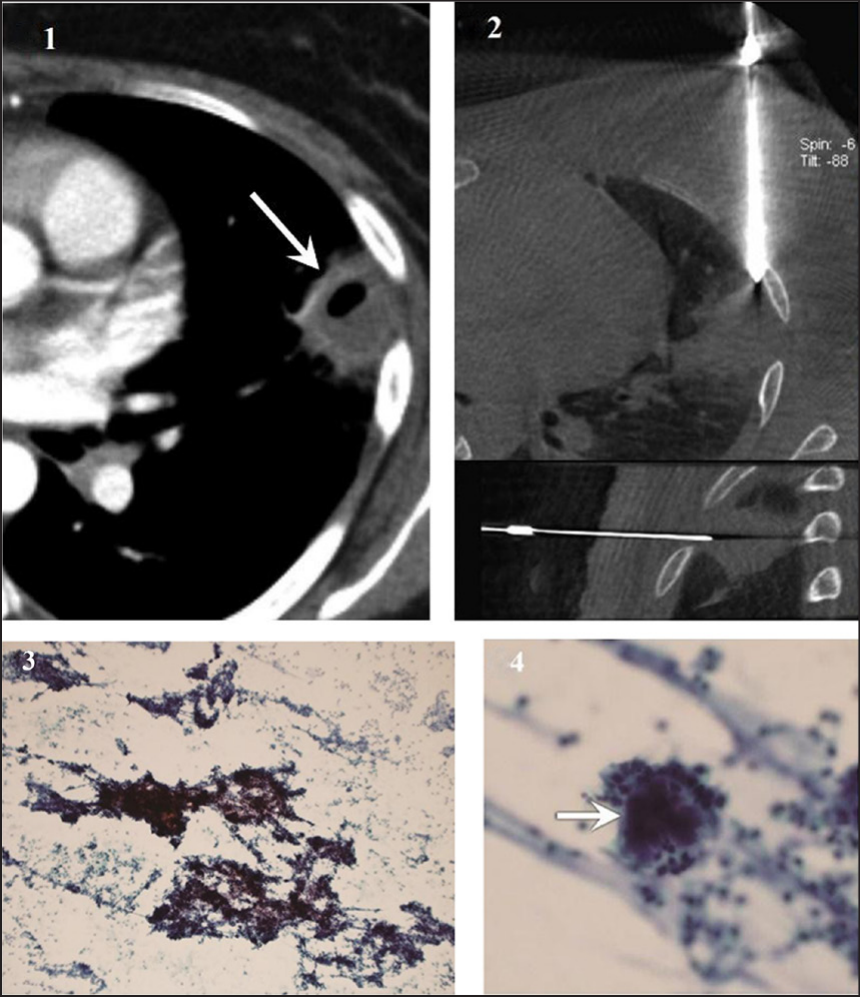
Actinomycosis diagnosed using samples from fine needle aspiration. **(1)** CT image shows a subpleural cavitary consolidation in the left upper lobe (arrow). **(2)** Cone-beam CT-guided CT images (upper, axial; lower, sagittal) show coaxial guide-needle placement to the target lesion. **(3)** Photomicrograph of cytological smear (Papanicolaou stain, original magnification × 100) shows clumps of basophilic bacterial colonies admixed with neutrophilic suppurative inflammatory infiltrates **(4)** Magnified photomicrograph of cytological smear (Papanicolaou stain, original magnification × 400) shows sulfur granules with dense center (arrow) surrounded by delicate filaments. The left upper lobe lesion disappeared after appropriate antibiotic therapy.

Among the procedures in which an adequate sample was obtained from both FNA and CNB, 72 were benign. Forty-eight cases were benign-specific lesions. FNA diagnosed benign lesions as benign-specific lesions in 23 cases (23/72, 31.9%). CNB diagnosed benign lesions as benign-specific lesions in 46 cases (46/72, 63.9%). The rate of benign-specific lesion diagnosis was significantly higher for CNB than for FNA (63.9% vs. 31.9%, *p* < 0.001). Among 49 cases of FNA showing benign-nonspecific lesions, CNB showed benign-specific lesions in 25 cases (25/49, 51.0%). Among 26 cases of CNB showing benign-nonspecific lesions, FNA showed benign-specific lesions in 2 cases (2/26, 7.7%) (Figure 4, Table 4).

**Table 4:** Results of FNA and CNB in the diagnosis of benign-specific lesions.

Final diagnosis	No. (%) of final diagnosis by FNA	No. (%) of final diagnosis by CNB

Tuberculosis (n = 24)	Tuberculosis, 10 (41.7%)	Tuberculosis, 24 (100%)
	Benign-nonspecific lesion, 14 (58.3%)	
Fungal infection (n = 11)	Fungal infection, 7 (63.6%) (Aspergillosis 4, *Cryptococcus* 1, *Pneumocystis jirovecii* 1, nonspecific fungal infection 1)	Fungal infection, 11 (100%) (Aspergillosis 4, *Cryptococcus* 4, Histoplasmosis 1, *Pneumocystis jirovecii* 1, nonspecific fungal infection 1)
	Benign-nonspecific lesion, 4	
Actinomycosis (n = 4)	Actinomycosis, 4 (100%)	Actinomycosis, 2 (50%)
		Benign-nonspecific lesion, 2 (50%)
Hamartoma (n = 4)	Hamartoma, 1 (25%)	Hamartoma, 4 (100%)
	Benign-nonspecific lesion, 3 (75%)	

*Note.* FNA = fine needle aspiration, CNB = core needle biopsy. Lesions in which both FNA and CNB showed adequate samples are shown. Final diagnoses with fewer than 3 cases are not shown.

Pneumothorax occurred in 50 (16.7%, 50/300) procedures. Chest tube insertion was required in 3 patients (1%, 3/300). Hemoptysis occurred in 18 (6.0%, 18/300) procedures. Five days after biopsies, one patient died of arrhythmia attributable to cardiac involvement of eosinophilic granulomatosis with polyangiitis.

## Discussion

We assessed the diagnostic outcomes of the combination of FNA and CNB using the same coaxial guide-needle under CBCT guidance of intrathoracic lesions. A lesion underwent both CNB and FNA in the same session. Then we compared FNA and CNB in the procedures in which both showed adequate samples.

In the present study, similar to the results of previous studies, the use of sequential FNA and CNB increased the rate of an adequate specimen and diagnostic accuracy, compared to FNA or CNB alone [4,5,6,7,8,9]. These previous studies compared same-session sequential FNA and CNB of intrathoracic lesions under CT guidance [4,5,7,8,9] or CT-fluoroscopic guidance [6]. The present study evaluated same-session sequential FNA and CNB under CBCT guidance. The rates of adequate specimens and diagnostic accuracy of the malignant lesion by combined use of CNB and FNA were 97.6% and 100%, respectively, in the present study, compared to 99.6% and 97.0%, respectively, in a study using CBCT guidance CNB [16].

In the present study, the hemoptysis rate of 6.0% is in the range (1.1%–6.5%) of those of same-session sequential FNA and CNB under CT guidance [4,7,9] and CT-fluoroscopic guidance [6]. It is similar to that of CBCT-guided CNB (6.9%) [16].

In the present study, the pneumothorax rate of 16.7% was lower than that of most of the other studies of sequential FNA and CNB in the same procedure, where the pneumothorax rate ranged from 18% to 54% [4,6,7,8,9]. The lower rate of pneumothorax in the present study may be the result of using the same coaxial guide-needle for FNA and CNB in order to reduce the number of pleural punctures. Boiselle’s et al. study, which used the same coaxial guide-needle, showed 18% pneumothorax [7]. In other studies, when the same coaxial needle was not used in the sequential procedure, the pneumothorax rate was 24–32.6% [4,6,9]. However, despite using the same coaxial needle technique, the pneumothorax rate was 54% in an earlier study which used CT guidance biopsy [8].

Our study differs from other studies in its guidance; we used CBCT-guided biopsy with virtual guidance in all cases. In CBCT-guided biopsies, including our study, when patients undergo CBCT, the three-dimensional CT images are reconstructed using multiplanar reformations in the axial, coronal, and sagittal planes, as appropriate [16,17,19,20,21,22]. Ohno’s et al. study, using CT or CT-fluoroscopic guidance, showed that, compared with the conventional method, the multiplanar reconstruction method significantly improved both success rates and diagnostic accuracy without a significant increase in pneumothorax rate [23]. In another study, virtual guidance was a significant protective factor against pneumothorax in CBCT-guided percutaneous transthoracic needle biopsy [16].

Other studies compared the results of CNB and FNA in intrathoracic lesions and reported that CNB showed better diagnostic accuracy for benign lesions; however, these studies showed different results for malignant lesions. In some cases, no significant difference existed between FNA and CNB for malignant lesions [5,6,8,13]; in others, FNA was better than CNB for malignant lesions [4,7,9]; in still others, CNB was better than FNA [10,11,12,24]. In our study, when we included atypical cells in malignancy, FNA and CNB did not show a significant difference in the diagnosis of malignancy. However, when atypical cells were considered benign, CNB showed significantly higher diagnostic accuracy than FNA. We suggest that atypical cells should be regarded as benign in the assessment of the diagnostic accuracy of biopsy, because a result of atypical cells requires additional FNA, CNB, or surgical resection to rule out lung cancer [1].

In the present study, as well as earlier studies, the rate for the specific diagnosis of malignancy was significantly higher for CNB than for FNA [11,13]. CNB is more accurate for the diagnosis of lymphoma [9,25]. Gong et al. showed that FNA and CNB had similar diagnostic accuracy for malignant epithelial neoplasms, but CNB had better diagnostic accuracy than FNA for nonepithelial malignant neoplasms [5]. The differences in the performance of CNB compared to FNA may be because of the different ratio of subgroups of malignancy in the previous studies. In the present study, however, most of the malignant lesions were epithelial neoplasms.

In the present study, the rate of benign-specific lesions was significantly higher for CNB than for FNA (63.9% vs. 31.9%). The other studies also found that CNB demonstrates a greater ability than FNA to determine a specific diagnosis for benign lesions [8,13]. Findings of benign-nonspecific lesions including no malignant cells require long-term follow-up or repeated biopsy, as a finding of a benign-nonspecific lesion may be caused by sampling error [26]. One study shows that, on follow up, 10.6% of the nonspecific-benign biopsy results were a missed tumor [27]. Our result indicates CNB is better for the diagnosis of benign lesions and patient management.

In the present study, the rate of adequate samples was higher for CNB than for FNA. However, there was a difference in biopsy procedures compared to previous studies. We performed CNB first, followed by FNA. In most of the other studies that compared same-session sequential FNA and CNB, FNA was performed first [4,5,6,7,9]. If bleeding occurred in the tissue, whichever procedure is performed first could affect the result of the second procedure. In addition, Klein et al. reported that the coaxial needle tip moved, due to patient motion or pneumothorax after the first procedure [8]. In our study, a cytopathologist was not present during the procedure. In other studies, comparing FNA and CNB, an on-site cytopathologist immediately assessed the aspirate specimen, and additional aspirates or CNB were obtained if the aspirate specimen was considered inadequate [4,5,7,9,10]. The diagnostic accuracy of FNA is reduced in the absence of an on-site cytopathologist [28]. Choi et al. showed that aspiration method alone was an independent risk factor associated with diagnostic failure in CT-guided CNB or FNA [29]. Choi et al. also did not have an on-site cytopathologist.

In the present study, FNA showed better results than CNB in six cases. Minot’s et al. analysis of false negative FNA or CNB, showed that necrotic lesions could cause interpretative errors, because of paucicellularity of rare tumor cells [26]. In the study by Bocking et al., 7 out of 16 cases of hemorrhagic infarction by biopsy or aspiration turned out to be a carcinoma [13]. These results are consistent with our results in which malignancies showed infarct or predominant necrotic cells and inflammatory exudate respectively in CNB. Similarly, in two cases, actinomycosis was specifically diagnosed by FNA; but abscess was diagnosed by CNB.

There are several limitations to the present study. First, it is a retrospective study. The pathologist may not have interpreted the FNA and CNB results separately and may have been aware of the FNA results when interpreting the CNB specimen. Second, the absence of an on-site cytopathologist may cause lower performance of FNA, as described above. Third, CNB is more likely to yield sufficient tissue for mutation analysis [1,11], although that is beyond the scope of this study.

## Conclusion

The combined use of CNB and FNA using the same coaxial guide-needle under CBCT guidance showed better diagnostic performance than CNB or FNA alone. In the comparison of CNB and FNA, CNB showed significantly better performance.

## Abbreviations

- Fine needle aspiration biopsy (FNA)
- Core needle biopsy (CNB)
- C-arm cone-beam CT system (CBCT)
